# Transforming Duchenne muscular dystrophy therapy: The multifaceted role of extracellular vesicles and exosomes

**DOI:** 10.1016/j.bbrep.2026.102578

**Published:** 2026-04-13

**Authors:** Fatemeh Farzi, Fatemeh Soltanmohammadi, Seyed Abolghasem Mohammadi, Nosratollah Zarghami, Effat Alizadeh

**Affiliations:** a Department of Medical Biotechnology, Faculty of Advanced Medical Sciences, Tabriz University of Medical Sciences, Tabriz, Iran; b Department of Pharmaceutics, Faculty of Pharmacy, Tabriz University of Medical Sciences, Tabriz, Iran; c Student Research Committee, Tabriz University of Medical Sciences, Tabriz, Iran; d Department of Plant Breeding and Biotechnology, University of Tabriz, Tabriz, Iran; e Center of Excellence for Cereal Molecular Breeding, University of Tabriz, Tabriz, Iran; f Department of Clinical Biochemistry, Faculty of Medicine, Istanbul Aydin University, Istanbul, Turkey; g Physical Medicine and Rehabilitation Research Center, Tabriz University of Medical Sciences, Tabriz, Iran

**Keywords:** Duchenne muscular dystrophy (DMD), Extracellular vesicles (EVs), Exosomes, Cell/gene therapy

## Abstract

Duchenne muscular dystrophy (DMD) remains a devastating X chromosome‐linked disorder with restricted curative options. Additionally, the existing treatment approaches, such as growth-modulating agents, anti-inflammatory drugs, antisense oligonucleotides with exon-skipping capabilities, stop codon mutation suppressors, vector-mediated gene therapy, CRISPR/Cas9 gene editing, and exogenous cell transplantation, can delay disease progression but are not curative. Extracellular vesicles (EVs), especially exosomes, nanoscale vesicles involved in intercellular communication, have emerged as promising therapeutic tools for DMD due to their low immunogenicity, ability to deliver therapeutic cargos, and potential to modulate inflammation, oxidative stress, and fibrosis. This review explores the transformative role of EVs (including exosomes) as multifunctional tools in DMD management. Natural EVs, enriched with regenerative microRNAs (miRNAs) and anti-fibrotic proteins, modulate inflammation, oxidative stress, and muscle degeneration. Besides, innovative engineering approaches could improve EVs' cargo loading and targeting, assisting efficient delivery of oligonucleotides and CRISPR/Cas9 editing components. Furthermore, we address the capacity of these vesicles to restore dystrophin expression and attenuate pathogenic mechanisms. Lastly, challenges associated with EV isolation, stability, and scalability are critically evaluated to support the development of an integrated cell and gene therapy framework with significant potential to improve clinical outcomes in DMD patients.

## Highlights

• Exosomes emerged as natural agents having therapeutic potential for various disease.
• Exosomes carry non-coding RNAs and proteins that help inhibit muscle fibrosis.
• Exosomes can deliver CRISPR/Cas9, enabling its packaging and prolonged circulation.

## 1 Introduction

The most prevalent type of muscular dystrophy (MD) is a form with X-linked inheritance and early childhood onset, known as DMD [1]. Mutations in the dystrophin gene lead to skeletal muscle degeneration and result in fibrotic tissue replacement [2]. The heart muscle is one of the most crucial muscles that can cause disabilities for individualswith limb-girdle, facioscapulohumeral, Duchenne, and Becker-Emery-Dreifuss [3] MD. Since 10-15% of people with MD die due to cardiac disorders [4], early detection and attenuation of the heart failure symptoms are vital [5]. DMD can be caused by either a spontaneous mutation or an inherited nonsense point mutation [6]. This disease is characterized by progressive muscle weakness, wasting, and ultimately death.

The DMD gene consists of 79 exons and encodes a protein comprising 3685 amino acids, organized into four major domains [7,8].These domains interact with various proteins and complexes, and their insufficiency results in sarcolemmal weakening, elevated free radical production and cytosolic calcium levels, ultimately leading to impaired muscle regeneration in DMD models. [7]. These factors contribute to myofiber damage, chronic inflammation, progressive fibrosis, and dysfunction of muscle stem cells (MuSCs). Due to its size, the DMD gene is prone to various types of mutations, including large mutations (79%), deletions (68%), duplications (11%), and small mutations such as nonsense mutations (10%), deletions (5%), insertions (2%), and splice site mutations (3%) [8]. As individuals with DMD can live up to their third decade of life, both they and their families face significant physical, emotional, and financial burden [9]. Therefore, scientists must continue their efforts to find effective treatments for this disease. Since 1988, research in this field has focused on various therapeutic strategies, including corticosteroids [10], growth-modulating agents, anti-inflammatory drugs [11], antisense oligonucleotides with exon-skipping capabilities [12,13], stop codon mutation suppressors [14], vector-mediated gene therapy [15], CRISPR/Cas9 gene editing [16], and exogenous cell transplantation [17]. Each of these strategies targets a specific type of DMD mutation. For example, eteplirsen, golodirsen, and viltolarsen have received FDA approval for the treatment of patients with exon-skipping type DMD [[12], [13], [14],^18^]. However, these treatments are not curative and can only delay the onset or slow down the progression of the disease. Additionally, each of these strategies has its own limitations. For example, gene therapy by adenoviral vectors causes immune responses in patients [19]. Unexpected changes in the genome may occur as a result of CRISPR/Cas9 off-target effects, leading to unintended alterations in non-target genomic regions. [20]. Antisense oligonucleotides with exon-skipping capabilities are effective in treating only approximately 50% of patients with DMD.

[18]. In light of these challenges, cell-based therapy may be a promising avenue for DMD treatment. However, cell therapy is associated with the risk of tumor genesis, low engraftment rate, and low viability of transplanted cells [21]. Extracellular vesicles (EVs) are lipid-bound vesicles secreted by cells into the extracellular space. The three subtypes of EVs are microvesicles (MVs), exosomes, and other types [22]. These subtypes are categorized according to their biogenesis and release pathways, size, content, and functionality [23]. Exosomes and MVs have been widely used as carriers of biomolecules, drugs, and genes. Moreover, these EVs, especially those obtained from biofluids, are a rich source of disease biomarkers, making them potential tools for diseases diagnosis [23,24]. Exosomes are released by all cell types and are involved in intercellular communication by transferring nucleic acids, proteins, lipids, amino acids, and other metabolites to distant cells [24,25]. This unique ability makes them a potential therapeutic tool for various diseases. Modified exosomes can be designed to deliver therapeutic cargos, such as interfering RNA, antisense oligonucleotides, chemotherapeutic agents, and immunomodulators, to specific targets [24]. Exosomes have several advantages, including a low risk of infusion-related toxicity, immunogenicity, and tumorigenic potential. Given the high mortality rate and the prevalence of cardiomyopathy in DMD, it is a promising candidate for exosome-based therapy [26]. In this review, we will discuss the therapeutic potential of EVs and exosomes and their current usage in terms of DMD treatment, with a particular focus on their role in disease progression.

## 2 EVs and soluble factors in conditioned media

### 2.1 Extracellular vesicles (EVs)

**Fig. 1:**
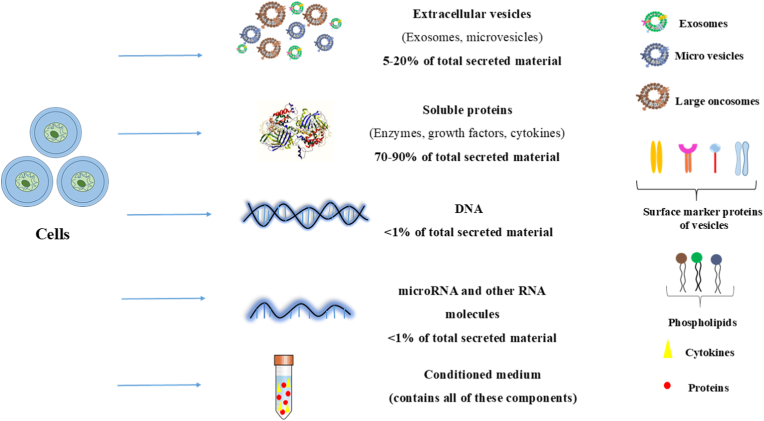
Th secretome of cells has several items components, including extracellular vesicles, growth factors, DNA, microRNA, and other factors, which could be used for cell-free treatments. Conditioned medium, which is secreted by all cell types, is composed of proteins, cytokines, growth factors, chemokines, lipids, and non-coding RNAs.

We want to focus on the therapeutic benefits of EVs present in the secretome (Fig. 1). EVs are classified as a heterogeneous group of lipid bilayer–enclosed particles released by cells that cannot replicate and lack a functional nucleus. According to the MISEV 2018/2023 guidelines, EVs should be primarily described by physical characteristics, such as size, density, or biochemical markers, rather than by assumed biogenesis pathways, unless experimentally demonstrated [22,27]. These EVs can be subdivided based on size (e.g., small EVs <200 nm, medium/large EVs >200 nm), density, or surface markers. Direct classification into exosomes or microvesicles should be avoided unless biogenesis has been experimentally confirmed. These vesicles contain a variety of proteins, lipids, metabolites, and nucleic acids.

**Fig. 2:**
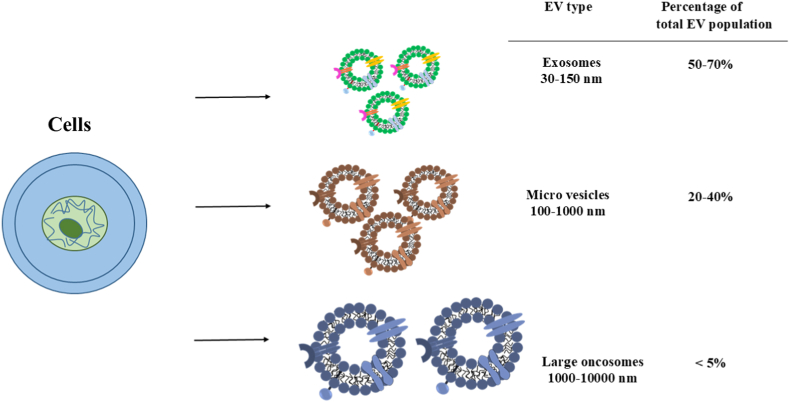
There are 4 types of cell-secreted vesicles, including exosomes, microvesicles, and large oncosomes, which are different in size. Also, the contents of vesicles are specific to cell type and have several surface markers and signaling molecules which affect their therapeutic potential.

As shown in Fig. 2, EVs can include small EVs, medium/large EVs, and large oncosome-like vesicles. However, the terms “exosomes,” “microvesicles,” and “oncosomes” are used only when mechanistic evidence supports their origin [28,29].

#### 2.1.1 Micro vesicles (MVs) or ectosomes

The MISEV guidelines recommend avoiding assigning EVs to the “microvesicle” category unless outward budding from the plasma membrane has been experimentally demonstrated**.** Operationally, these vesicles may be categorized as medium/large EVs (typically >200 nm) when classified solely on the basis of size analysis. Their cargo may include proteins, nucleic acids, and lipids [30]. Although these vesicles have therapeutic potential, the lack of standardized, biogenesis-confirming isolation methods means that most studies should refer to them simply as medium/large EVs [22]. Standardization challenges, cargo heterogeneity, and isolation-related impurities continue to limit clinical use. EVs are formed by direct outward budding from the plasma membrane [31]. As these particles act like exosomes, using them is expected to have a wide range of therapeutic potential. However, the existence of some obstacles apparently makes it hard for scientists to reach this beneficial potency. One of these obstacles is the limitation in medium size MV's isolation techniques. Cell debris or lipoproteins are common impurities that can result from most isolation techniques. Moreover, medium size MVs are extremely variable in size and molecular content, making their standardization and therapeutic utilization sophisticated. The optimization of them for clinical use is hindered by the incomplete investigation of their biological functions and mechanisms [32]. Despite the obstacles, medium size MV-based treatment has made remarkable progress, currently with a focus on cardiovascular diseases. These particles have been shown to increase in quantity in coagulation and vascular-related problems by inducing platelet adhesion and fibrin deposition on atherosclerotic arteries. Medium size MVs also induce platelet adhesion to collagen surfaces. Considering all these factors, circulating medium size MVs can provide a potential non-invasive biomarker to predict the severity of cardiovascular diseases [33]. But so far, there has been no study in the field of treating muscular diseases with medium size MVs.

#### 2.1.2 Oncosomes

Oncosomes were initially described in cancer models; however, MISEV2023 emphasizes that “large oncosomes” or “oncosome-like EVs” should only be used when their oncogenic origin and size range (1000-10,000 nm in diameter) have been experimentally validated. A study on human glioblastoma in 2008 led to the discovery of a new subgroup of EVs. [34,35]. These EVs can carry oncogenic proteins, DNA, RNA, and other bioactive molecules. In this review, we refer to them as large EVs enriched in oncogenic cargo unless mechanistic data confirm oncogenic biogenesis. They are involved in intercellular communication in the tumor microenvironment, enabling procedures such as tumor progression, metastasis, and drug resistance [34,35]. The oncosomes or oncosome-like EVs present in the blood and other biofluids have been used for cancer diagnosis, monitoring cancer progression, and treatment response [36,37]. These inimitable features led to the classification of these EVs as large oncosomes (LOs)/oncosome-like EVs. However, one year after their discovery, large oncosomes were recognized as a distinct group with unique morphology, structure, and content, contrary to the initial belief that they were merely enlarged forms of regular oncosomes. [35]. LOs have been used in liquid biopsies to diagnose and monitor cancer progression, especially in prostate cancer. Inhibiting LO or oncosome generation or uptake can interrupt tumor-supportive signaling pathways, suggesting a novel therapeutic approach [38].

#### 2.1.3 Exosomes

The MISEV discourages defining EVs as exosomes purely based on size (30–150 nm) or presumed endosomal origin unless specific markers and mechanistic evidence (e.g., Endosomal Sorting Complex Required for Transport (ESCRT) dependence) are provided. To maintain rigor, we refer to these nanoparticles as small EVs (<200 nm), reserving the term “exosomes” for those with experimentally confirmed endosomal origin [22]. Small EVs are surrounded by a lipid bilayer with a size range of 30-150 nm and were first defined in 1981. These nano vesicles are classified into natural and modified small EVs. Modified small EVs are exosomes that have been surface-modified, loaded with drugs or biologic molecules, or subjected with other bioengineering procedures. Their development aims to improve the targeting capability, drug delivery efficiency, and therapeutic effects of natural exosomes in numerous diseases, like cancer, neurological disorders, and autoimmune diseases. [[38], [39], [40]]. In the second step of classification, natural small EVs are divided into animal-derived and plant-derived small EVs. Finally, animal-derived small EVs can be further categorized as normal or tumor derived [41]. Nearly all cell types secrete exosomes under normal conditions, but this process has been reported to have a faster turnover in certain situations, such as tumor growth, metastasis, and immune regulation. However, from another perspective, these elements may have other benefits and serve other roles, such as acting as a diagnostic marker or monitoring disease progression. Additionally, small EVs have the potential to treat various diseases, including those related to muscles [42,43]. Both modified and natural small EVs have been used for this purpose [43]. For instance, it has been shown that exosomes can transfer a high amount of the miR-29 family, which takes part in muscle differentiation and inhibits tissue fibrosis [44]. Additionally, treatment with exosomes, which were harvested from murine differentiated myotubes, has been shown to improve the membrane integrity and muscle function in mdx mice (with DMD) [42]. The use of a protein known as proprotein by Ran and coworkers, resulted in the inhibition of myostatin and the enhancement of muscle regeneration and growth in MDX mice. [45]. Proprotein was anchored to the exosomes' surface, leading to an increase in its serum stability. Moreover, modified exosomes have been developed for targeting the IL6 *trans*-signaling pathway and expressing IL6 signal transducer (IL6ST) decoy receptor on the surface of exosomes in order to inhibit chronic inflammation in muscle pathophysiology [46]. Functionally, IL6ST-exosomes blocked IL-6/sIL-6R/induced STAT3 phosphorylation in reporter cells, without altering classical pathway activation by IL-6. In vitro and in vivo studies have been performed. In C2C12 myoblast cells, IL6ST-exosomes treatment reduced phosphorylated STAT3 levels and partially restored myogenic differentiation markers such as MyoD and Myogenin. In vivo IL6ST-exosomes systemically administered (**1×10^10^** EVs per injection, biweekly for 4 weeks in the mdx mouse model of DMD decreased STAT3 phosphorylation by ∼50% in quadriceps and gastrocnemius muscles. Also, moderately amended muscle fiber morphology [46].

### 2.2 Conditioned media

The upper liquid collected from cultured cells is called conditioned media, and it contains secreted factors that can be used for therapeutic purposes [47]. Conditioned medium can be secreted from various cell-types and is composed of proteins, cytokines, GFs, chemokines, lipids, and non-coding RNAs [48,49].

#### 2.2.1 Growth factors

GFs are a noteworthy kind of cell-free therapy. These are signaling proteins secreted by cells that regulate numerous biological processes, namely cell proliferation, differentiation, migration, and tissue repair. Various types of GFs, such as platelet-derived GF, epidermal GF, fibroblast GF, and transforming GF-beta, are extensively utilized in regenerative medicine and therapeutic applications [26,50,51].

#### 2.2.2 Micro-RNAs

The miRNA is a small, non-coding RNA molecule, typically 18–25 nucleotides in length, that modulate gene expression at the post-transcriptional level. They can be isolated from multiple kinds of cells and have been used for regenerative and therapeutic purposes [52,53].

## 3 Molecular pathology of DMD and emerging roles of EVs

### 3.1 Pathobiology of DMD

DMD is a severe recessive neuromuscular disorder with X-linked inheritance initiated by mutations in the dystrophin gene. Dystrophin, as a large cytoskeletal protein, is essential for maintaining sarcolemmal stability. Also, the dystrophin-associated glycoprotein complex serves as a link between the cytoskeleton and the extracellular matrix. [54]. The absence of functional dystrophin reduces muscle fibers and causes susceptibility to contraction-induced damage, leading to repeated cycles of necrosis and regeneration [55]. With prolonged disease progression, the regenerative potential gradually declines, ultimately leading to extensive replacement of muscle tissue by fibrotic and adipose components The pathological course mentioned is additionally intensified by chronic inflammation, oxidative stress, and dysregulated calcium homeostasis, which together increase muscle degeneration and damage satellite cell functions. Such disturbances, both at the molecular and cellular level, finally convert to the progressive muscle fault and other DMD complications, including cardiomyopathy as well as respiratory failure [54].

### 3.2 The role of exosomes and EVs in the pathogenesis of DMD

It is becoming evident from the investigations that EVs and exosomes may have a role in the pathology of DMD. According to the fact that miRNAs are the main component of EVs and exosomes, changes in the levels of these miRNAs are correlated with the pathologic processes in the dystrophic muscles, including fibrosis, inflammation, and cardiomyopathy. For instance, Yedigaryan et al. confirmed that expression of miR-206, an effective modulator of collagen synthesis, especially in fibrogenic recipient cells, was upregulated in the EVs of dystrophic mice. This process is associated with fibrotic pathogenesis resulting from impaired satellite cell function, which leads to deficient muscle regeneration.

Aswad et al. demonstrated that high concentrations of palmitate and saturated fatty acids in obese mice elevate intramuscular ceramide levels, which are known to modulate EV secretion from muscle [56]. Furthermore, it has been reported that an inhibitor of EX release and ceramide synthesis is capable of refining muscle function in mdx mice [57]. These findings support the idea of the existence of destructive organ cross-talks linked to the circulation of detrimental EV and EX contents.

Studies have indicated that small EVs enriched from skeletal muscle–conditioned media can be taken up by cardiac tissue following injection into mice [56]. The term “exosomes” is avoided because the study did not demonstrate endosomal biogenesis. On the other hand, exosomes obtained from CDCs have been observed to be taken up by skeletal muscles [58]. Additionally, research suggests that miRNAs released from the myocardium during the development of heart failure may have negative effects on skeletal muscle [59]. This highlights a potential role of EVs and exosomes secreted in dystrophic muscles, contributing to ongoing muscle degeneration. This occurs through the interaction between EVs/exosomes from skeletal and cardiac muscle, as the progression of cardiomyopathy and the dissociation of skeletal muscle in DMD appear to facilitate the exchange of harmful EVs/exosomes between the two tissues, ultimately worsening the disease. Skeletal and cardiac muscle communicate through EVs/exosomes.

It has been confirmed that changes in the levels of miRNAs in exosomes or EVs of DMD patients are an efficient biomarker for the diagnosis of DMD. Exosomal miRNAs such as miR-206, miR-1, miR-133A, miR-133B, and miR-199a-5p that are reported to be involved in tissue fibrosis, muscle wasting, and myogenic cell proliferation serve as potential biomarkers for DMD diagnosis [[60], [61], [62]]. Studies have demonstrated that EV extracted from DMD patients' plasma is an effective biomarker for DMD. Plasma-EV-derived miR-133a-3p was upregulated in DMD patients compared to healthy individuals. However, the amount of the expression of plasma-EV-derived miR-29c-3p was downregulated in DMD subjects compared to healthy participants. Moreover, studies have confirmed that certain categories of surface antigens on EVs are linked to the expression of miRNAs in DMD patients. For example, Matsuzaka et al. confirmed that EVs associated with CD63 and major histocompatibility complex (MHC) II, obtained from the serum of DMD patients, exhibited a high enrichment of muscle-abundant miRNAs in comparison to healthy participants, particularly miR-1, miR-133a, and miR-206 in CD63-associated EVs, and miR-1 and miR-133a in MHC II-associated EVs [57].

In summary, skeletal muscle–derived EVs and exosomes in DMD models require further investigation to elucidate their role in disease pathogenesis and to support the development of new EV-based diagnostic and therapeutic strategies. Table 1. Study-level evidence map for EV-based interventions in Duchenne muscular dystrophy (MISEV2023-aligned).

**Table 1:** Evidence-mapping and study-level appraisal of included EV studies in DMD.

Study/Reference	Study Design & Model	EV Source & Isolation	EV Characterization (MISEV2023)	Intervention/Engineering Strategy	Efficacy Outcomes	Mechanistic Evidence	Methodological Limitations/Risk of Bias	Off Target Effects(#)
**PL-MSC exosome study** [44]	In vitro myoblasts + *mdx* mice	Placenta-derived MSCs; small EV fraction	Prior studies show CD63/CD81/Alix, TEM, NTA— but not repeated here	Native exosomes, systemic/local injection	↑ dystrophin, ↓ TGF-β, ↓ fibrosis (heart, diaphragm)	Exosome miR-29c can cause good effects	Markers confirmation and purity is missing	Off-target organ uptake of exosomes through systemic administration
**FAP-EVs after HDAC inhibitor** [63]	In vitro MuSC assays + *mdx* mice	FAPs; EV isolation not fully described	Characterization not reported	Native EVs with pharmacologically altered cargo	↑ MuSC activation, ↓ fibrosis/inflammation in *mdx*	Strong causal link: ↑ miR-206 in EVs, loss-of-function shown	Missing EV purification details	Off-target effects of HDAC inhibitor
**CDC-EV study** [58]	*mdx* mice	Cardiosphere-derived cells; isolation done in original papers	Well-characterized historically; but markers not restated	Native CDC-exosomes injected intramyocardially	↑ LV function, ↑ running capacity; ↓ oxidative stress	Linked to miR-148a but not loss-of-function tested	No marker panel and unclear EVs levels	Possible immunogenicity of human CDC exsosomes in mice
**Myogenic progenitor cell exosomes** [64]	*mdx* mice	Myogenic progenitors; isolation not described	No markers, no size distribution	Native exosomes	Anti-inflammatory effects; improved cardiac molecular markers	No mechanistic validation	Absence of EV isolation &characterization, high MISEV risk	Probable Off target effects of systemic delivery/short time transcriptome effects
**DMD cardiac exosomes** [43,65]	DMD iPSC-derived cardiomyocytes	DMD-cardiomyocyte cultures; EV isolation assumed	Cargo profiled (miRNAs) but no EV protein markers	Native disease-derived EVs	↑ ROS, ↑ apoptosis, impaired survival	Strong causal evidence: miR-339-5p depletion rescues phenotype	Missing EV-depleted control; unclear purity	Possible pharmacologic off-target confusing/in vivo safety/biodistribution reporting
**EAA/CP05-PMO engineered EVs]** [66,67]	In vitro uptake + *mdx* mouse injections	Myotube-derived exosomes; aptamer anchoring	Need confirmation of integrity post-modification	Engineered EVs: PMO anchored via CP05/EAA aptamer	↑ uptake, ↑ dystrophin + fibers in *mdx*	Causality assumed; no RNase protection or lysis controls	Encapsulation vs surface adsorption unresolved	Possible immunogenicity of EAA
**Serum EVs delivering Crisper Cas9** [68]	*mdx* mice genome editing	Serum EVs; loading method unclear	No confirmation of EV-Cas9 loading efficiency	EV-CRISPR: SpCas9 protein delivered inside EVs	Exon 24–25 deletion; dystrophin restoration	Could be free Cas9 contamination	High risk of overclaim without EV-free control	Probably off target genome effects
**Engineered EVs for transient CRISPR** [69]	In vitro + reporter mice + *mdx* mice	Engineered EVs from modified producer cells	Likely characterized in original ref; not included here	Cas9 recruited with FKBP–FRB system; sgRNA tethered by viral motifs	High exon-skipping efficiency; long-lasting correction	Mechanism consistent but requires free-RNP exclusion	Needs EV integrity, purity, and control arms	May off-target mutagenesis/immunogenicity

#: In several studies reported in Table 1, off-target effects were not evaluated, accordingly, statements about off-target risk were authors' perspective except supported by experiments. PL-MSC: Placenta derived mesenchymal stem cells, FAPs: Fibro-adipogenic progenitors. HDAC: Histone deacetylase. DMD: Duchenne muscular dystrophy. MuSCs: muscle stem cells. EVs: Extracellular Vesicles. EAA: Exosomal Anchor DNA Aptamer. PMOs: Phosphorodiamidate morpholino oligomers. RNP: Ribonucleo protein. sgRNA: single guide RNA. CDCs: Cardiosphere-derived cells. LV: Left ventricular myocardium.

## 4 EVs and exosomes for the treatment of DMD

### 4.1 Un-modified EVs in the treatment of DMD

**Fig. 3:**
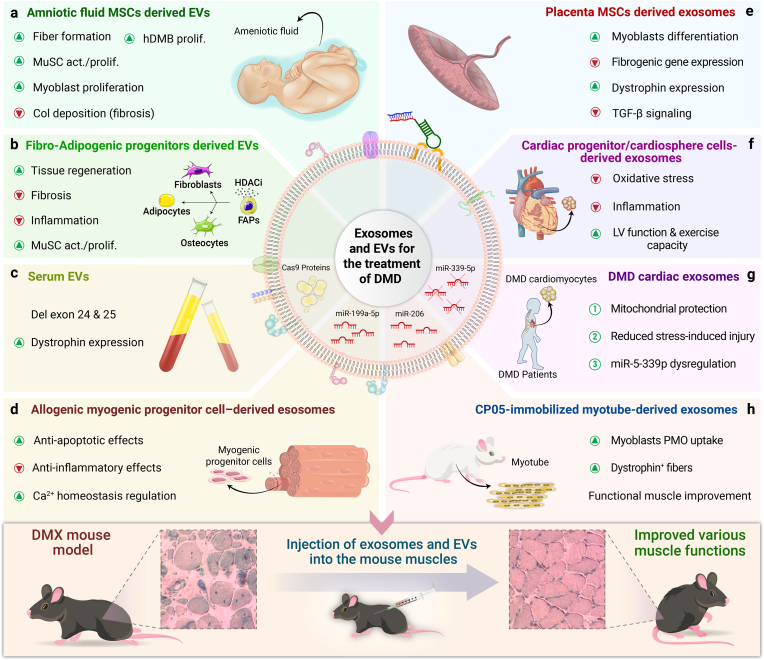
Researches on applications of EVs and exosomes to treat DMD. a) Amniotic fluid MSCs-derived EVs, b) Fibro-Adipogenic progenitors-derived EVs, c) serum EVs, d) Allogenic Myogenic Progenitor cells derived exosomes, e) Placenta MSCs derived exosomes, f) Cardiac progenitor/cardiosphere cells derived exosomes, g) DMD cardiac exosomes, and h) CP05 immobilized myotube derived exosomes. EVs: Extracellular Vesicles. DMD: Duchenne Muscular Dystrophy; MuSCs: muscle stem cells. Del: deletion. LV: Left ventricular myocardium. PMOs: Phosphorodiamidate morpholino oligomers. MSCs: Mesenchymal stem cells activation (act), ca2+: calcium. Molecular effects include increased dystrophin (DMD) expression suppression of fibrogenic signaling (TGFB1), regulation of apoptosis-related genes (BAX, BCL2), modulation of ca2+handling genes (RYR1, ATP2A1), and disease-associated microRNAs such as miR-339-5p. In the DMX mouse model, administration of selected exosomes/EVs improves muscle structure and function. Col: collagen. HDMB: human dystrophic myoblast. Prolif: proliferation. DMD-ICMs: DMD cardiomyocytes. CP05: cholesterol or exosomal anchor peptide.

Mesenchymal stem cells (MSCs) have been widely used for tissue regeneration [70]. It is worth mentioning that the beneficial effect of MSCs was attributed to the paracrine effect rather than their differentiation ability [71]. The EVs released from MSCs have been reported to be involved in muscle regeneration [72]. Thus, Sandona and co-workers isolated EVs from amniotic fluid MSCs (AFMSCs) and investigated their efficiency in DMD treatment [73].Mutations in patients with DMD impair the ability of MuSCs to differentiate. This ultimately results in the depletion of the skeletal muscle's ability to regenerate [74]. EVs derived from AFMSCs enhanced the proliferation of human dystrophic myoblasts and mouse dystrophic MuSCs. Furthermore, local administration of these EVs in mdx mice led to new fiber formation, a higher number of activated MuSCs, and decreased collagen deposition.(Fig. 3a).

### 4.2 Modified EVs for the treatment of DMD

Naturally derived EVs may serve as promising therapeutic agents in their unmodified state; however, modified EVs exhibit superior therapeutic efficacy. This is attributed to their low probability of inducing an immune response and their capacity for modification to deliver beneficial cargo and target particular cell types [24,75]. Thus, the content of EVs can be modified by two main procedures, namely endogenous or exogenous. To achieve this endogenously, it is necessary to induce the source cell to overexpress the specified cargo, whether it is mRNA, miRNA, or protein. Subsequently, by means of diffusion, the EVs that are released from that modified or modified cell will inherently possess a greater concentration of that substance as their cargo. Modification of the content of EVs post-release from the source cell necessitates the application of physical or chemical methods. The methods encompass incubating EVs with the intended cargo, employing electroporation, utilizing sonication, etc [40,75].

#### 4.2.1 Modified EVs for non-dystrophin restoration DMD treatment

**Fig. 4:**
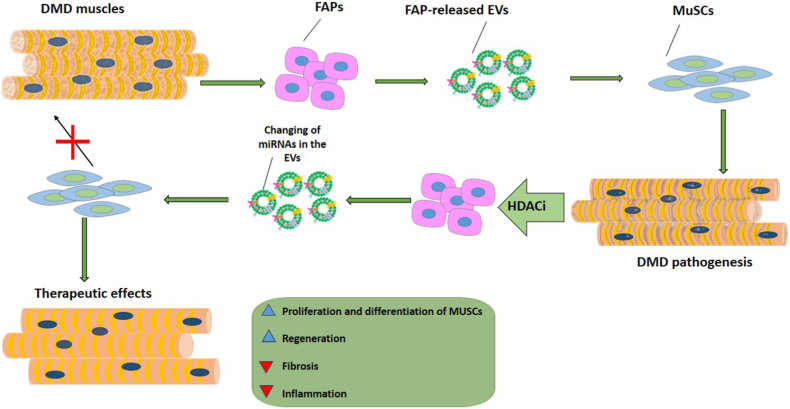
EVs released by FAPs of DMD muscles exposed to HDAC inhibitors have therapeutic effects against DMD, namely reduction in the development of fibrosis and inflammation in dystrophic muscles, and stimulating tissue regeneration. This is related to the changes in the microRNA content of EVs. EVs (extracellular vesicles); FAPs (fibro–adipogenic progenitors); DMD (Duchenne Muscular Dystrophy); MuSCs (muscle stem cells).

Modified EVs can be utilized for the more efficient treatment of DMD. For instance, a study showed that fibro–adipogenic progenitors (FAPs) release EVs that facilitate the transfer of miRNAs to MuSCs [63] (Fig. 3b). FAPs are identified as crucial cellular targets of histone deacetylase inhibitors (HDACi), which are pharmacological compounds used to counteract the progression of DMD by stimulating compensatory regeneration [76]. When dystrophic MSCs were exposed to HDACis, the levels of certain miRNAs inside EVs increased. These miRNAs work together to target biological processes that are important for DMD therapy, such as regeneration, fibrosis, and inflammation (Fig. 4). Elevated concentrations of miR-206 in EVs generated by FAPs in the muscles of patients with DMD or mdx animals treated with HDACi are linked to improved tissue regeneration and reduced fibrosis. Consistently, EVs derived from HDACi-treated dystrophic FAPs were able to enhance the activation and proliferation of MuSCs in vitro, as well as stimulate tissue regeneration. Furthermore, when these EVs were transplanted directly into the muscles of mdx mice, they effectively suppressed the development of fibrosis and inflammation in dystrophic muscles (Fig. 4). The use of AntagomiR to block individual miRNAs demonstrated that miR-206 is specifically required for the development of MuSCs and the regeneration of dystrophic muscles produced by EVs. Additionally, it suggests that the combined activity of miRNAs generated by HDACi is responsible for the overall biological action of these EVs. These observations indicate that manipulating the content of EVs through pharmacological means could be a new approach for therapeutic interventions in MD (Table 2).

**Table 2:** Overview of key points related to EV applications in DMD treatment.

Section	Key points	Refs
**Naturally Derived EVs for DMD Treatment**	- **The role of MSCs:** MSCs are effective for tissue regeneration due to paracrine effects rather than differentiation. Isolated EVs from AFMSCs showed efficacy in DMD treatment. - **Impact of DMD Mutations:** Mutations hinder MuSC differentiation, depleting muscle regeneration ability. - **AFMSCs-derived EVs:** Increased proliferation of dystrophic myoblasts/MuSCs, new fiber formation, more activated MuSCs, and reduced collagen deposition in mdx mice.	[71], [73], [74]
**Modified EVs for DMD Treatment**	- **Advantages of modified EVs:** Superior therapeutic efficacy due to lower immune response and modification capabilities. - **Modification methods:** Endogenous (overexpressing cargo in source cells) and exogenous (physical/chemical methods post-release). - **FAPs and miRNAs:** FAPs release EVs, transferring miRNAs to MuSCs, targeting regeneration, fibrosis, and inflammation. - **The role of miR-206:** Essential for MuSC development and muscle regeneration; elevated in HDACi-treated FAPs.	[40,75], [63]
**Gene Therapy with EVs for DMD Treatment**	- **Use of EVs as gene therapy vectors:** Suggests using the Cas9 protein instead of the gene to minimize immune responses. Isolation of serum EVs encapsulating SpCas9 protein; effectively deleted exons 24 and 25 in mdx mice, leading to dystrophin expression. - **Transient delivery systems:** Ribonucleoprotein delivery of CRISPR-Cas9 reduces off-target effects.	[77]

EVs: extracellular vesicles, DMD: Duchenne muscular dystrophy, FAPs: fibro–adipogenic progenitors.

#### 4.2.2 Modified EVs for restoring dystrophin expression

EVs have been widely used as vectors of gene therapy [78]. Using the Cas9 protein rather than the Cas9 gene is suggested to reduce immune responses and unintended mutations associated with CRISPR/Cas9-based gene editing. [79].Consequently, Majeau et al. isolated serum EVs and encapsulated SpCas9 protein within EVs [77]. This approach effectively excised exons 24 and 25 in mdx mice, thereby restoring dystrophin expression.(Fig. 3c).

The extended expression of the CRISPR-Cas9 nuclease and gRNA via viral vectors can lead to off-target mutations and potential immunogenic responses. Thus, a transient delivery system is required for therapeutic genome editing applications. Ribonucleoprotein delivery of CRISPR-Cas9 possesses numerous benefits over DNA delivery. It enables potent on-target cleavage while also decreasing undesirable off-target effects. Gee et al. developed a transient delivery system utilizing EVs derived from HEK293T cells [69]. They achieved this through the use of two separate homing mechanismsThe first method involved chemical-induced dimerization, which facilitated the recruitment of Cas9 protein into EVs. The Cas9 protein was incorporated into EVs in producer cells by the FKBP12 and FRB dimerization system. The FRB variant (T2098L) binds the rapamycin analog (AP21967) with high affinity. The interaction was subsequently exploited to selectively load Cas9 protein into EVs budding from producer cells.The second homing mechanism was a viral RNA packaging signal and two self-cleaving riboswitches. They could facilitate the tethering and release of sgRNA into EVs. The sgRNA is reported to be localized in the nucleus. Conversely, to be able to load it in EVs, sgRNA should be transferred into the cytoplasm and localized near budding EVs for efficacious packaging in producer cells. Thereby, they constructed an expression vector with two lentiviral vector components, the Tat activation response element (TAR) in the 5′ LTR promoter region and an extended Psi (Ψ+) packaging signal that specifically binds to nucleocapsid of Gag, to express mRNA containing an AmCyan-coding sequence. Additionally, to release packaged mRNA from the designed delivery system after inoculation into target cells, they flanked the sgRNA with a self-cleaving ribozyme. Based on the results, genome editing efficiency was demonstrated in several challenging cell types, such as human induced pluripotent stem (iPS) cells, neurons, and myoblasts. The developed genome-editing delivery system achieved exon-skipping efficiencies exceeding 90% in skeletal muscle cells derived from iPSCs of DMD patients. Furthermore, a single injection of the designed genome editing system resulted in long-lasting genomic exon skipping in both a luciferase reporter mouse and mdx mice.

Existing studies on EV-mediated CRISPR/Cas9 delivery for DMD treatment remain primarily at the preclinical stage and exhibit varying levels of practical quality. Maximum evidence originates from in vitro experiments directed at myoblasts or limited animal models (e.g., mdx mice), exhibiting inadequate reproducibility across laboratories. Key restrictions include efficiency and specificity. CRISPR/Cas9 loading of EVs is variable, and editing potential in muscle tissue remains unperfected.

The tropism of EVs for skeletal and cardiac muscle is not yet optimized, resulting in inconsistent biodistribution and delivery. Enduring immune responses, off-target effects, and the scalability of EVs needto be thoroughly evaluated and understood. Standardization and potency assays are necessary to address translational gaps.Therefore, CRISPR/Cas9 delivery using EVs is not yet therapeutically ready. Additional work is essential to certify delivery efficiency, safety, reproducibility, and clinical translation.

EVs can also be used to encapsulate adeno-associated viruses (AAVs) to reduce their immunogenicity.Encapsulating the AAV vector within EVs, which are naturally produced as biological carriers, may reduce the host immune response to the vector. This approach can be used for the treatment of DMD through gene therapy [80]. Nevertheless, to date, this approach has not been investigated for the treatment of DMD. (Table 2).

### 4.3 Un-modified exosomes in the treatment of DMD

Small EV-associated miRNAs can serve as biomarkers for diagnosing DMD. MISEV recommends biomarker attribution to EV-associated cargo, not to exosomes, unless a definitive origin is proven [22]. Studies have shown that the levels of miR-206, miR-1, miR-133A, and miR-133B, which are encapsulated in circulating exosomes in the blood, hold promise as biomarkers for monitoring the progression of muscular dystrophy in patients [60]. Moreover, exosomes derived from DMD fibroblasts are rich in miR-199a-5p. This miRNA has been reported to be overexpressed in the fibrotic tissues. Additionally, treating fibroblasts with miR-199a-5p increases the expression of α-smooth muscle actin (α-SMA) and collagen, markers of the myofibroblast phenotype and tissue fibrosis [81].

Exosomes can not only serve as a bio-marker for the diagnosis of MD, but can also be utilized as a therapeutic agent against MD progression. For example, a study used exosomes derived from allogeneic myogenic progenitor cells to modulate gene expression in the mdx mouse model. [82]. The *Bcl-2* gene was found to be upregulated following exosome therapy, indicating the anti-apoptotic effect of exosomes. In contrast, the expression levels of *Wnt5a*, *Map K8*, and *IL-6* were decreased, suggesting the anti-inflammatory effect of exosome therapy. These genes are associated with chronic inflammation, the progression of heart failure, and the pathogenesis of cardiac disease. Additionally, the *ATP2B4* and *VDR* genes exhibited upregulation in exosomes-treated mdx mice, indicating their crucial role in cellular calcium ion regulation (Fig. 3d). Altogether, these observations confirm the efficacy of exosome therapy in improving heart function in DMD patients.

Exosomes are responsible for the therapeutic paracrine effects of MSCs in various diseases [26,83]. In light of this observation, Ariel Bier et al. conducted a study on the therapeutic effect of small EVs enriched from placenta-derived MSCs (PL-MSCs) in DMD [44]. The pre-treatment of myoblasts with exosomes induced their differentiation and inhibited the expression of fibrogenic genes in these cells. This was consistent with an increase in the expression of ubiquitous dystrophin and decreased expression of TGF-β. The most notable therapeutic impact in DMD animal models was attributed to miR-29c [84]. These researchers reported that miR-29c was transferred from PL-MSCs to myoblasts during co-culture. Furthermore, PL-MSCs significantly reduced the expression of TGF-β and the extent of fibrosis in the diaphragm and heart muscles, suppressed inflammation, and enhanced ubiquitous dystrophin expression (Fig. 3e). The results suggest that PL-MSCs and their released exosomes have promising clinical uses in the cell treatment of DMD, partially through the specific delivery of exosomal miR-29c [79].

Accordingly, researchers have shifted their focus towards the use of cell-derived exosomes for treating DMD, rather than using cells themselves. Several studies have confirmed the potential of exosomes in treating DMD patients. For example, in one study, researchers employed cardiac progenitor/cardiosphere cells (CDCs) to treat DMD in mdx mice [85]. Following intramyocardial injection of CDCs or CDC-derived exosomes into mdx mice, improvements were observed in left ventricular function, ambulatory function, and exercise capacity, along with reduced oxidative stress and suppressed inflammation.(Fig. 3f). Furthermore, intramyocardial injection of either CDCs or CDCs-derived exosomes also led to transient expression of dystrophin in the heart, diaphragm, and soleus of mdx mice. When exosomes were injected into the left ventricle, they were distributed to various organs, including the heart, skeletal muscle, brain, liver, lung, spleen, gut, and kidneys of mdx mice. The injection of exosomes into the skeletal muscle resulted in enhanced myofiber number, increased proliferation, and increased levels of the myogenin transcription factor, which is responsible for myoblast differentiation [86]. Finally, it was explored that exosomal miR-148a, which is responsible for enhanced dystrophin protein expression, mediates the beneficial effects of CDCs or CDCs-derived exosomes in DMD patients.

Gartz et al. explored the response of DMD cardiomyocytes (DMD-iCMs) to long-term exposure to DMD cardiac exosomes [43]. DMD-iCMs exhibited stress susceptibility, as evidenced by elevated ROS levels and increased cell mortality. Prolonged exposure to non-affected exosomes had a protective effect. Conversely, prolonged exposure to DMD cardiac exosomes did not provide protection, and the stress response was enhanced by inhibiting the production of DMD cardiac exosomes both in vivo and in vitro. Furthermore, it was found that reducing the endogenous release of DMD cardiac exosomes prevents stress-induced injury in DMD-iCMs. DMD cardiac exosomes exposure also resulted in the regulation of cGMP-PKG and TGF-β pathways by stimulating transcriptional changes, both of which are related to DMD. The pathogenic consequences of DMD-exo were attributed to the miRNA content rather than the exosome surface peptides. To examine the impact of exosomal miRNA, DMD-iCMs were treated with exosomes enriched with miRNAs or miRNA-depleted exosomes, and then injury was induced by stress. MiRNA-depleted exosomes reduced ROS levels in DMD-iCMs compared to miRNA-enriched exosomes. The same group studied the process by which DMD cardiac exosomes hinder the cellular response by modifying crucial stress-responsive genes in the recipient cells [65].They provided evidence that DMD-iCMs release exosomes with altered miRNA profiles compared to healthy controls. Specifically, miR-339-5p showed increased expression in DMD-iCMs, DMD exosomes, and mdx mice cardiac tissue.Restoring dystrophin in DMD-iCMs enhanced stress responsiveness and correlated with downregulation of miR-339-5p, indicating its specificity to DMD pathology. Suppression of miR-339-5p led to increased expression of MDM2, GSK3A, and MAP2K3, genes involved in stress-responsive signaling pathways (Fig. 3g). Ultimately, inhibition of miR-339-5p protected mitochondria and reduced cell death in DMD-iCMs, suggesting that miR-339-5p directly regulates the stress response. These findings suggest that exosomal miR-339-5p might serve as a significant target specific to the disease for future therapeutic breakthroughs in the treatment and diagnosis of DMD cardiomyopathy.

### 4.4 Modified exosomes in the treatment of DMD

#### 4.4.1 Modified exosomes for non-dystrophin restoration DMD treatment

As mentioned previously, naturally derived exosomes can be utilized for the treatment of DMD. However, the application of these exosomes is limited due to their low targeting ability. To overcome this problem, modified exosomes have been developed. Modified exosomes can be generated by pre-treating parent cells with drugs or other agents, through genetic manipulation, or by optimizing culture conditions. Another approach involves modifying naturally derived exosomes by loading them with desired cargo, altering their surface, or developing exosome-nanoparticle hybrid systems. [38,39,87]. Villa et al. attached annexin A1 (ANXA1) to the surface of myoblast cell-derived exosomes (ANX-exosomes) [88]. ANXA1 has been found to prevent inflammatory responses in the body and induce the polarization of macrophages toward an anti-inflammatory phenotype [[89], [90], [91]]. They immobilized ANX-exosomes on the prepared hydrated magnesium silicate nanotubes that were covered by ferromagnetic nanoparticles (Mag-ANX-exosomes). As a result, after injection of Mag-ANX-exosomes using a magnetic field, the prepared delivery system successfully localized itself in the skeletal muscles. They found that attaching ANXA1 to the exosomes resulted in an increased level of anti-inflammatory M2-associated genes. In addition, intramuscular injection of ANX-exosomes into the tibialis anterior muscle of mdx mice led to their uptake by immune cells and localized enrichment within the tissue, resulting in macrophage immunomodulation and enhanced muscle force. To evaluate the targeting ability of the designed nanoplatform, Mag-ANX-exosomes or ANX-exosomes were injected into mdx mice. In the absence of a magnetic field, both of the injections led to the accumulation of Mag-ANX-exosomes and ANX-exosomes in the liver, spleen, muscles, lungs, and intestines. In the presence of a magnetic field, a stronger MRI signal was seen in the muscles of Mag-ANX-exosomes-injected mdx mice compared to that in PBS-injected mdx mice. This confirms that the localization of the developed delivery system in the muscles is due to the presence of a magnetic field. This study highlights the importance of designing modified exosomes to improve their biodistribution and enhance their efficacy in DMD treatment.

#### 4.4.2 Modified exosomes for dystrophin restoration

Exosomes have shown potential as carriers for drug and gene delivery, in addition to their inherent beneficial effects related to their cargo. Oligonucleotides, which include short single- or double-stranded DNA or RNA, have various subgroups such as antisense oligonucleotides (ASOs), RNA interference, miRNAs, and aptamers [92]. Oligonucleotides can inhibit a specific gene expression. However, the clinical application of oligonucleotides is limited due to their rapid renal clearance, poor membrane permeability, and nuclease-induced degradation [[93], [94], [95]]. To date, peptide vectors [96], nano carriers [97], bioconjugates [98], viral vectors [99,100], and exosomes have been used for oligonucleotide delivery. Exosomes have several advantages for delivering oligonucleotides. They can easily cross biological membranes [101]. CD47 protein on the exosome's surface inhibits its phagocytosis, which in turn increases its circulation time compared to liposomes [102]. Exosomes are considered safe and can be isolated in a way that matches the individual's own cells. Exosomes from specific sources possess inherent properties that promote tissue regeneration and reduce inflammation, thereby enhancing the efficacy of therapeutic oligonucleotide delivery. [103,104]. Oligonucleotides have been loaded into exosomes using two main approaches: encapsulation and surface modification. The low loading efficiency has been reported to restrict the efficiency of the encapsulation method [105,106]. Considering these factors, Han and colleagues screened a library of single-stranded DNA aptamers using systematic evolution of ligands by exponential enrichment (SELEX). [66]. They targeted CP05-immobilized murine myotube-derived exosomes.

(Fig. 3h) and successfully found a DNA aptamer that specifically binds to exosomes, which they designated exosomal anchor aptamer (EAA). EAA exhibited high binding affinity to exosomes and facilitated the DMD phosphorodiamidate morpholino oligomer (DMD-PMO) on murine myotube-derived exosomes (exosome-EAA-PMO) through annealing or direct synthesis. PMO is an antisense oligonucleotide against murine DMD exon 23 [67]. In vivo experiments revealed that exosome-EAA-PMO could achieve significantly higher uptake by mouse myoblasts compared to PMO. Exosome-EAA-PMO also led to enhanced dystrophin-positive fiber percentage following intramuscular injection, compared to PMO. Furthermore, exosomes amplified the efficiency of PMO activities by improving their distribution to the muscle. This led to the production of therapeutic amounts of dystrophin and functional improvement in mdx mice. This study opened up a new avenue for loading oligonucleotides into exosomes, providing a promising approach for clinical application in DMD management.

A quantitative summary of Exosome–EAA–PMO is summarized in Table 3.

**Table 3:** Quantitative summary of exosomal anchor aptamer (EAA)in DMD phosphorodiamidate morpholino oligomer.

Parameter	PMO Alone	Exosome–EAA–PMO	Relative Change
Cellular uptake (myoblasts)	Baseline	↑ Significant	>1 × increase
Dystrophin-positive fibers	Lower	Higher	↑ Enhanced restoration
Muscle distribution efficiency	Limited	Improved	↑ Efficiency

Additionally, the investigations indicate the potential for administering an mRNA that encodes a full-length dystrophin for localized and eventually systemic treatment of DMD [66,107]. Therefore, Saleem et al. conducted a study in which they encapsulated full-length dystrophin mRNA into the exosomes (mRNA@exosomes) [108]. Following intravascular injection of mRNA@exosomes into mdx mice, Western blot and immunohistochemistry analyses showed that the treatment effectively reduced serum creatine kinase levels, improved grip strength, and alleviated muscle hypertrophy. Furthermore, western blotting and immunohistochemistry analysis revealed the expression of dystrophin protein in both skeletal and cardiac muscles. Looking at it in its entirety, these investigations shed light on the fact that exosomes can serve as vectors for the delivery of OA, mRNAs, or other gene therapy agents for the treatment of DMD.

## 5 Challenges of using EVs and exosomes in clinical settings

The benefits of using EVs/exosomes as gene delivery vectors over other vectors have been summarized in Table 4. Notwithstanding these benefits, their application is constrained by certain restrictions.

**Table 4:** Comparing the gene delivery ability of EVs, exosomes, AAVs, and LNPs.

Feature	EVs	Exosomes	AAVs	LNPs	Refs
**Origin**	Natural	Natural	Viral	Synthetic	[101,109,110]
**Immunogenicity**	Very low	Very low	High	Moderate	[[111], [112], [113]]
**Targeting specificity**	Moderate (EVs have inherent targeting ability. Additionally, EVs can be modified with targeting agents to attain specificity)	High (Inherent targeting dependent on cell type of origin, engineered Exosomes can be designed to attain targeting ability)	High (tissue serotype dependent)	Should be modified to attain targeting ability	[[114], [115], [116], [117], [118], [119]]
**Gene expression duration**	Transient	Transient	Long term	Transient	[[120], [121], [122]]
**Safety**	High	High	Low	Moderate	[[111], [112], [113]]
**Scalability**	Challenging	Challenging	Complex and expensive	Scalable	[[123], [124], [125]]

EVs: extracellular vesicles, LNPs: lipid nanocarriers, AAVs: adeno-associated viral vectors.

A primary difficulty is identifying a secure source that yields efficient exosomes/EVs with reduced immunogenicity [126]. The method of isolation can influence the outcomes of clinical interventions, as well as the concentration, purity, and size of exosomes/EVs. For example, miRNA profiles vary based on the isolation techniques employed [127]. This problem is heightened due to the ambiguity of the instructions in the International Society of Extracellular Vesicles (ISEV) standards, which permit researchers to use discretion in the isolation of exosomes/EVs [27].

Moreover, a significant challenge is that the ideal circumstances for cell culture conflict with those required for exosomes/EVs isolation. Consequently, both optimal circumstances for cellular proliferation and appropriate parameters for exosome extraction must be meticulously determined [128]. The passage number of cells in the passage may influence the cargo and efficacy of the exosomes/EVs. In elevated passage numbers, the efficacy of the acquired exosomes/EVs may diminish [129]. The donor's age is an additional factor in assessing the efficacy of exosomes/EVs. Exosomes/EVs obtained from older donors exhibit diminished effectiveness [130]. The quantity of seeded cells can influence the function and content of exosomes/EVs. Consequently, employing a consistent seeding process is crucial for ensuring reproducibility [129]. In addition, exosomes/EVs are disposed to aggregation and degradation, resulting in the loss of cargo and decreased functionality over time [131]. Differences in isolation procedures of exosomes/EVs can cause inconsistent cargo retention and purity, affecting their therapeutic potential [132]. Challenges in ensuring the stability of isolated exosomes/EVs during storage can reduce the integrity of their cargo [133]. Another limitation is the low cargo loading efficiency of exosomes/EVs.

Furthermore, independent of the administration mode and cellular origin, systemically injected exosomes/EVs are rapidly eliminated from the body by macrophages of the reticuloendothelial system (RES) [134]. Fewer than 5% of the administered exosomes/EVs dosage has been found to persist in the bloodstream 3 h post-injection [135]. Nonetheless, natural and synthetic hydrogels can carry a limited quantity of exosomes/EVs, while effectively producing the intended therapeutic effect and sustaining it for a specified duration. Hydrogels can inhibit the premature clearance of encapsulated exosomes/EVs [136].

Despite extensive research on exosome/EV-based therapies, clinical translation faces major challenges, including limitations in isolation and purification methods, a lack of reproducible protocols for producing well-characterized and homogeneous exosomes, and insufficient data on exosome stability. The absence of scalability and inadequate validation of isolation methods remain significant obstacles to the clinical translation of exosomes/EVs, which is crucial for large-scale manufacturing. The microfluidic exosome isolation method remains in the early stages of research. Despite its significant potential, poor yield remains the primary constraint, necessitating additional attempts for enhancement [123].

Another challenge limiting the clinical application of EVs and exosomes is batch-to-batch variability. Research has shown that anti-inflammatory properties, production yield, therapeutic efficacy, size distribution, and purity vary between EVs from different batches, highlighting challenges in their reproducibility. [[137], [138], [139]]. Exosomes/EVs have been reported to deliver oncogenic factors, including miRNAs, to target cells, modulating immune responses and the tumor microenvironment, which facilitates tumor proliferation and metastasis to adjacent tissues [140,141].

The other challenge that restricts the clinical application of exosomes/EVs is long-term storage. The physicochemical characteristics and protein content are two important factors often assessed in the research of exosomes/EVs stability. Unfortunately, the majority of research focuses solely on the effects of temperature on exosome stability, with inconsistent findings. Additionally, storage conditions influence the cargo, size, and structural integrity of exosomes/EVs. [123]. The storage temperature and duration significantly affect the recovery yield and morphology of exosomes/EVs, with temperatures below −70 °C being optimal for preserving newly isolated exosomes/EVs for clinical applications [142]. After 4 weeks of storage at −20 and 4 °C, it has been observed that the particle size of EVs increases while the protein content decreases. It is noteworthy that EVs maintain stability at a neutral pH (6.8–7.4), but harsh pH conditions can cause their rapid degradation.Additionally, it should be noted that freeze-thaw cycles can cause the vesicular membranes to rupture, leading to the release of cargo and aggregation of vesicles [139]. Nonetheless, the preservation of the properties and integrity of exosomes/EVs during long-term storage is essential and poses a significant challenge [143]. On the other hand, EVs possess a short-term hypercoagulable effect and may be involved in the early phase of post-injury hemostasis. However, the exact role of EVs in trauma-induced coagulopathy and posttraumatic thrombosis should be explored [144].

Appearance, osmolality, subvisible particles, particle number, protein content, total protein, purity, host cell protein and DNA, and sterility are the factors that should be assessed for quality control of EVs/exosomes [145]. The commercialization of exosomes/EVs formulations necessitates strict good manufacturing practices (GMP) processes for their production, encompassing exosomes/EVs source selection, standardized cell culture methodologies, downstream exosomes/EVs isolation, and quality evaluation protocols. A GMP-grade exosome/EV production procedure comprises the type of cells, culture environment, cultivation system, dissociation enzyme, and culture medium. Post-production, further purification is necessary, generally carried out through a three-step procedure. The third issue in GMP of exosomes is the establishment of an identification technique, including physical structure and bioactivity function properties [146].

Another obstacle is that the variety of manufacturing methods complicates standardization, resulting in a disconnected regulatory framework. The existing global regulatory framework for exosomes/EVs can be categorized into two primary approaches: One line of research aims to characterize the constituent components, whereas the other investigates the physiological consequences of their secretion. Exosomes/EVs utilized as therapeutic agents should be regulated in a manner akin to biological pharmaceutical products. Exosomes/EVs, akin to biologics, have been examined for their size and protein composition. A pharmaceutical based on exosomes/EVs must receive clinical approval following an analysis of its molecule's composition and structure, as well as evidence of its pharmacokinetics and therapeutic efficiency. Nonetheless, illustrating the pharmacokinetics and therapeutic effectiveness of exosomes poses difficulties for regulatory authorities [147,148]. EV applications face several key issues that disturb consistency, safety, and regulatory progress. Primarily, batch-to-batch variability constitutes a significant challenge, with yields and purity varying by 20–60%, thereby compromising reproducibility. Employing standardized bioprocess controls can help minimize these inconsistencies.Another hurdle is rapid clearance, with less than 5% of EVs remaining in circulation, resulting in limited systemic bioavailability. Strategies, for instance, surface modification, may improve circulation duration. Exact dose normalization is also critical, since doses range from 10^9^ to 10^12^ particles, which often results in unpredictable efficacy. Furthermore, stringent quality control, especially adherence to GMP standards, is necessary, since a minimum purity of 3 × 10^10^ particles per microgram of protein is needed to prevent contaminants that may affect safety. In terms of scalability, yields of about 1–5 × 10^10^ particles by 10^6^ cells can limit production capacity, which hinders clinical translation. Optimizing the cell source can enhance output. Stability remains a key challenge; while storage at −70 °C maintains EV integrity, storage at −20 °C leads to a 30–40% loss, probably caused by cargo degradation. Utilizing cryoprotectants and reducing freeze–thaw cycles can mitigate these losses. In addition, the potential oncogenic risk persists, since tumor-derived EVs can carry miRNAs or Kras mutations that contribute to tumorigenesis. Hence, using non-tumorigenic, primary cell sources is crucial. Lastly, the clinical translation of EVs is hindered by regulatory challenges, given their classification as biologics according to FDA and EMA guidelines. Developing appropriate standards will be key to accelerating approval and confirming reliable therapeutic performance.

## 6 Author's perspective

In the author's view, the collective body of evidence strongly supports the notion that EVs, including exosomes, hold significant therapeutic potential for DMD. By carrying regulatory miRNAs, proteins, and signaling molecules capable of modulating inflammation, fibrosis, and muscle regeneration, and also, by excellent carrier properties to deliver exogenous curative cargos to control DMD.

Exosomes appear to act against several key pathological mechanisms simultaneously. This multi-target capacity, as well as their inherent biocompatibility and low immunogenicity, positions exosomes as an appealing alternative to traditional cell-based strategies.

However, the current literature also highlights substantial gaps that must be addressed before EVs or exosome-based interventions can move toward clinical application. These include the lack of standardized protocols for exosome isolation and characterization, limited understanding of their biodistribution and long-term safety, and insufficient consensus on effective dosing strategies. The author believes that future progress will depend heavily on the development of unified methodological frameworks, advanced exosome-engineering technologies, and more comprehensive in vivo studies focused specifically on DMD pathophysiology.

Overall, the author concludes that while exosome-based therapies are unlikely to serve as a stand-alone cure for DMD, their integration with contemporary approaches such as gene therapy, RNA-based therapeutics, or anti-fibrotic drugs may offer a more practical and synergistic path forward. The author anticipates that continued interdisciplinary research will be essential for transforming the conceptual promise of exosomes into clinically meaningful therapeutic options for patients with DMD.

## 7 Conclusion and future prospects

Exosomes and EVs are safe and effective therapeutic agents for DMD treatment due to their low probability of causing an immune response. In particular, when they are isolated from patients' own fluids or conditioned media of self-stem cells. They contain miRNAs and proteins that help inhibit tissue fibrosis. Exosome and EV therapy increases the production of dystrophin, reduces inflammation, decreases oxidative stress, and improves various muscle functions in DMD patients or mdx mice. These nanovesicles are a suitable carrier for oligonucleotides and the CRISPR/Cas 9 system that have been utilized for the treatment of DMD. It is probable to provide the possibility of combination therapy by loading both corticosteroid drugs and growth-modulating agents into the exosomes. The combination therapy is more efficient than monotherapy due to having beneficial effects through two or more different mechanisms.

The translational application of CRISPR/Cas9-based gene editing for prolonged DMD therapy is presently constrained by the activation of the immune system. Using exosomes to encapsulate the CRISPR/Cas9 gene editing tool offers a potential method to avoid immune system detection and reduce the probability of an immunological response [149]. Even though the CRISPR/Cas9 plasmid is too big to be enclosed by exosomes, there is a solution for this obstacle. Exosomes-liposome hybrid nanosystems have been effectively employed for the delivery of CRISPR/Cas9, enabling prolonged circulation and targeted delivery [150]. Moreover, exosomes can mediate cell-specific delivery of CRISPR/Cas9 through their surface proteins, minimizing off-target adverse effects of the gene editing system. [151]. Thus far, the application of exosome-liposome hybrid systems for CRISPR/Cas9 delivery in DMD therapy has not been investigated.

As mentioned previously, several exosomal miRNAs, including miR-206, miR-1, miR-133A, miR-133B, and miR-199a-5p, have been confirmed as biomarkers for MD diagnosis. These miRNAs mediate tissue fibrosis or promote the progression of muscle wasting in patients [60,81]. Thus, targeting these miRNAs can be a potential therapeutic approach for MD treatment.

Considering the overall context, it can be concluded that exosome and EV therapy have established a new direction to treat DMD disease. Besides, more research is needed to expand the therapeutic merits of DMD treatment methods based on exosomes and EVs.

## Consent to participate

Not applicable.

## Availability of data and material

This paper has no data.

## Consent for publication

Not applicable.

## Funding

Department of Medical Biotechnology, Faculty of Advanced Medical Sciences, 10.13039/501100004366Tabriz University of Medical Sciences, for supporting this study, grant number: 72667.D:\MYFILES\ELSEVIER\BBREP\00102578\PROOF\S-CEEDITING\gs1

## CRediT authorship contribution statement

**Fatemeh Farzi:** Data curation, Formal analysis, Investigation, Methodology, Resources, Software, Visualization, Writing – original draft. **Fatemeh Soltanmohammadi:** Data curation, Formal analysis, Investigation, Methodology, Resources, Software, Validation, Visualization, Writing – original draft, Writing – review & editing. **Seyed Abolghasem Mohammadi:** Data curation, Formal analysis, Investigation, Methodology, Resources, Software, Validation. **Nosratollah Zarghami:** Data curation, Formal analysis, Investigation, Methodology, Software, Supervision, Writing – original draft. **Effat Alizadeh:** Conceptualization, Data curation, Formal analysis, Funding acquisition, Investigation, Methodology, Project administration, Resources, Software, Supervision, Validation, Visualization, Writing – original draft, Writing – review & editing.

## Declaration of competing interest

The authors declare that they have no known competing financial interests or personal relationships that could have appeared to influence the work reported in this paper.

## Data Availability

No data was used for the research described in the article.
